# Retrieving sequences of enzymes experimentally characterized but erroneously annotated : the case of the putrescine carbamoyltransferase

**DOI:** 10.1186/1471-2164-5-52

**Published:** 2004-08-02

**Authors:** Daniil G Naumoff, Ying Xu, Nicolas Glansdorff, Bernard Labedan

**Affiliations:** 1 Institut de Génétique et Microbiologie, CNRS UMR 8621, Université Paris Sud, Bâtiment 409, 91405 Orsay Cedex, France; 2 Microbiology, Free University of Brussels (VUB) and J.M. Wiame Research Institute 1, ave E. Gryzon, B-1070, Brussels, Belgium; 3 State Institute for Genetics and Selection of Industrial Microorganisms I-Dorozhny proezd, 1, Moscow 117545, Russia

## Abstract

**Background:**

Annotating genomes remains an hazardous task. Mistakes or gaps in such a complex process may occur when relevant knowledge is ignored, whether lost, forgotten or overlooked. This paper exemplifies an approach which could help to ressucitate such meaningful data.

**Results:**

We show that a set of closely related sequences which have been annotated as ornithine carbamoyltransferases are actually putrescine carbamoyltransferases. This demonstration is based on the following points : (i) use of enzymatic data which had been overlooked, (ii) rediscovery of a short NH_2_-terminal sequence allowing to reannotate a wrongly annotated ornithine carbamoyltransferase as a putrescine carbamoyltransferase, (iii) identification of conserved motifs allowing to distinguish unambiguously between the two kinds of carbamoyltransferases, and (iv) comparative study of the gene context of these different sequences.

**Conclusions:**

We explain why this specific case of misannotation had not yet been described and draw attention to the fact that analogous instances must be rather frequent. We urge to be especially cautious when high sequence similarity is coupled with an apparent lack of biochemical information. Moreover, from the point of view of genome annotation, proteins which have been studied experimentally but are not correlated with sequence data in current databases qualify as "orphans", just as unassigned genomic open reading frames do. The strategy we used in this paper to bridge such gaps in knowledge could work whenever it is possible to collect a body of facts about experimental data, homology, unnoticed sequence data, and accurate informations about gene context.

## Background

As a consequence of the deluge of completely sequenced genomes belonging to a large array of species, one can expect to identify many homologues of enzymes which have been previously well studied at the experimental level. This seems to be the general rule and the public sequence databanks (DDBJ/EMBL/GenBank) are now inundated by putative amino acid sequences which have been annotated uniquely by the widely used two-step process : (1) detection of a homologous relationship by a pairwise sequence similarity search at the level of primary structure and (2) inference of functional similarity from this detected homology.

However, the opposite might be true. For various reasons one can either miss or misinterpret the actual function of a putative protein when annotating by homology, resulting in a wrong function transfer. Several studies have already emphasized this point (see, for example, [1-3]). On the other hand, beside these now well identified errors which are often due to automatic processes, more subtle mistakes may occur when some of the numerous effects of divergent evolution are overlooked. In particular, one of the insufficiently appreciated problems of functional assignment is that homologous proteins might catalyse different biochemical reactions. Here, we discuss an instance of erroneous annotation (misannotation) in genes of nitrogen metabolism which to our knowledge has not yet been brought up. We explain why this is so and draw attention to the fact that similar cases must actually be rather frequent.

## Results and Discussion

### Annotating distant carbamoyltransferases

Our group ([4,5]) is presently involved in deciphering the evolutionary relationships between two ubiquitous and essential proteins, aspartate carbamoyltransferase (ATCase, EC 2.1.3.2) which catalyses the first committed step of *de novo *pyrimidine biosynthesis and ornithine carbamoyltransferase (OTCase, EC 2.1.3.3) which plays a crucial role in both anabolism and catabolism of arginine.

**Figure 1:**
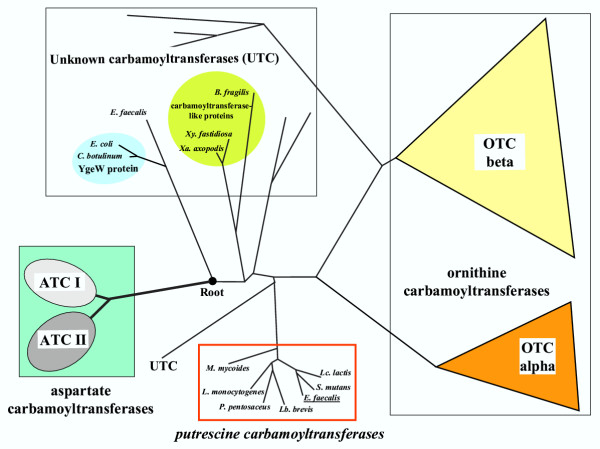
**A schematic evolutionary tree of carbamoyltransferases. **A distance tree of the 245 available ornithine carbamoyltransferases has been obtained using the PhyloTree programme [33]. This tree was rooted by a few paralogous aspartate carbamoyltransferases belonging to either family ATC I or ATC II ([4], [5]). The root is indicated by a closed circle. The two families OTC alpha and OTC beta are schematized as triangles. The homologous carbamoyltransferases which are branching far from these ATC or OTC families are labelled as unknown (UTC) even if some of them have been annotated as ornithine carbamoyltransferases (see text). The few UTC sequences which have been annotated as carbamoyltransferase-like are enclosed in ovals. The clade of reannotated putrescine carbamoyltransferases is framed in red.

In a recent study of the phylogeny of the 245 available OTCases (paper in preparation), we confirmed the existence of two families, OTC alpha and OTC beta, previously proposed on the basis of phylogenetic studies [4]. However, the advent of many new sequences further led to a more complex topology of the distance tree schematized on Fig. 1. It appears now that a significant number of sequences which have been annotated as OTCases are distantly related to either family as outlined on Fig. 1. These sequences, which are forming several clusters emerging in different locations between the root and the two families OTC alpha and OTC beta, have been provisionally annotated as UTC (unknown carbamoyltransferases). Indeed, among these UTC we found two sequences which do not correspond to classical OTCases.

The YgeW protein encoded by *Escherichia coli *and its close homologue from *Clostridium botulinum *are both located on a long branch emerging at the basis of this OTCases tree. YgeW is annotated as belonging to the ATCase/OTCase family (see, for example, the SwissProt knowledgebase [6]). On the branch which is next to the root we find the sequence of a protein which has been reported to be essential for arginine biosynthesis in the anaerobic bacterium *Bacteroides fragilis *[7]. This protein has been crystallized and characterized as a carbamoyltransferase-like protein since it does not display OTCase activity *in vitro *[7]. Indeed, several of its residues have been substituted in sites which are viewed as crucial for OTCase activity. Moreover, Dashuang *et al*. [7] indicated that a similar protein has been found in *Xylella fastidiosa*. Our phylogenetic data are in agreement with this observation since the protein annotated as OTCases in two strains of *X. fastidiosa *and its close relative present in two species of the *Xanthomonas *genus are found to branch close to that of *B. fragilis*. Therefore, the functional identification of these different UTC is certainly not straightforward and requires further investigations.

Furthermore, it occurred to us that, more than thirty years ago, another carbamoyltransferase was discovered by Roon and Barker [8]. A putrescine carbamoyltransferase (PTCase, EC 2.1.3.6) was found to be synthesized by the Gram-positive bacterium *Streptococcus *(now *Enterococcus*) *faecalis *when it was grown on agmatine but not arginine as primary energy source. This PTCase was easily separated from the OTCase synthesized by the same organism grown on arginine [8]. This putrescine carbamoyltransferase had further been studied by V. Stalon's group ([9-12]). Two features of this study – which had apparently been overlooked in recent genome annotations – appear now to be crucial for the interpretation of the data shown on Fig. 1. (1) Wargnies *et al*. [9] showed that the putrescine carbamoyltransferase of *E. faecalis *had a weak but unambiguous OTCase activity (7.4% in terms of Vmax, with K_M _for putrescine and L-ornithine of 0.029 mM and 13.0 mM respectively); (2) A short NH_2_-terminal sequence (29 residues) was published ten years later [13]. Since the complete genome of *E. faecalis *has now been sequenced [14], we could identify that one of the three putative ornithine carbamoyltransferases encoded by this genome, the open reading frame EF0732 annotated as ArgF-2 [14], is actually the putrescine carbamoyltransferase previously studied by the group of Stalon.

### A family of putrescine carbamoyltransferases

**Figure 2:**
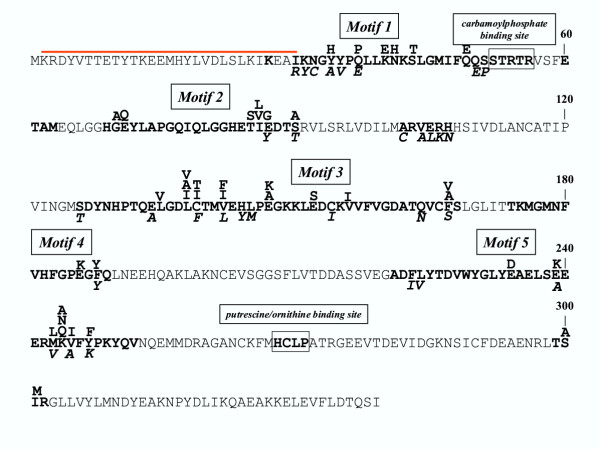
**Long motifs specific of putrescine carbamoyltransferases. **The amino acid sequence of the open reading frame EF0732 of *E. faecalis *annotated as ArgF-2 [14] but now reannotated as putrescine carbamoyltransferase is shown. The NH_2_-terminal 29 residues sequence [13] which helped to this reannotation is indicated by a red bar above the respective residues. The amino acids which are specifically conserved in putrescine carbamoyltransferases and not in ornithine carbamoyltransferases are printed in bold. Inside the long motifs (> 15 residues) found along the whole sequence and numbered as "Motif 1" to Motif 5"are indicated the few substitutions present in the other sequences (lines above the *E. faecalis *sequence) including those of *M. mycoides *which is more distant (line in italic below the *E. faecalis *sequence). The residues forming the respective binding sites of carbamoylphosphate and of either putrescine or ornithine are also in bold and framed.

In a second step, we extended this reannotation of a wrong OTCase as a PTCase to six other sequences encoded by *Lactococcus lactis*, *Streptococcus mutans, Pediococcus pentosaceus, Lactobacillus brevis *(and a very close partial sequence in *Lactobacillus sakei*) *, Listeria monocytogenes *and *Mycoplasma mycoides*, respectively. Indeed, these eight sequences, which have been annotated as either ArgF or ArcB (Table 1), (i) share high identity at the level of their amino acid sequence; (ii) they form a monophyletic group (Fig. 1) and (iii) they match the previously published *E. faecalis *NH_2_-terminal 29 residues sequence [13]. Moreover, as shown on Fig. 2, these sequences share several specific motifs which are not found in the homologous OTCases. These motifs, especially the five longer ones, are well conserved, even in *M. mycoides *which is however more distant. When these motifs are used together to query either the UniProt knowledgebase [15] or the nr (non-redundant) database using the PHI-Blast programme, we obtain only these putative PTCase sequences (including that of *M. mycoides*) to the exclusion of any other carbamoyltransferase.

**Table 1:** The seven ornithine carbamoyltransferases sequences which have been reannotated as putrescine carbamoyltransferases.

Species name	gene name	CDS	Swissprot TREMBL	Web link
*Enterococcus faecalis*	*argF-2*	EF0732	Q837U7	
*Lactococcus lactis*	*argF2*	LL1700	Q9CEY4	
*Streptococcus mutans*	*arcB*	SMU.262	Q8DW19	
*Pediococcus pentosaceus*^a^	-	Scaffold 18 Gene 459	-	
*Lactobacillus brevis*^a^	-	Scaffold 15 Gene 476	-	
*Lactobacillus sakei*^b^	*argF*	-	Q8RPX3	
*Listeria monocytogenes*	*arcB*	LMO0036	Q8YAS7	
*Mycoplasma mycoides*	*arcB*	MSC_0703	CAE77322	

^a ^: draft genome ^b ^: fragment

### Gene context, another tool for gene reannotation

**Figure 3:**
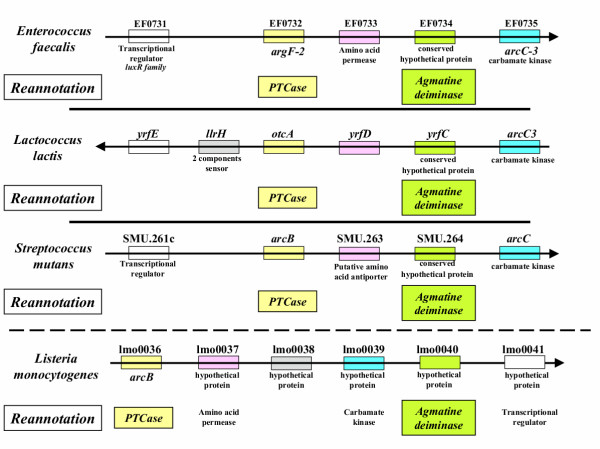
**Conservation of the gene context of putrescine carbamoyltransferases. **The open reading frame (ORF) EF0732 of *E. faecalis *is shown with neighbouring ORFs. Under each numbered ORF (EF0731 to EF0735) is indicated the putative function proposed by the annotators of this complete genome [14]. Below is the reannotation we are proposing. This schematic representation is repeated for the similar genomic regions present in three other completely sequenced genomes, those of *Lc. lactis*, *S. mutans *and *L. monocytogenes*. The homologous sequences are indicated by using the same color : white for transcriptional regulator, yellow for PTCase, pink for permease, green for agmatine deiminase, blue for carbamate kinase, respectively.

In a third step, the reannotation of this clade of OTCase sequences as PTCases was confirmed by a comparative study of the neighbourhood of their encoding genes present in the four genomes completely sequenced and published (*E. faecalis*, *Lc. lactis*, *S. mutans *and *L. monocytogenes*). As shown on Fig. 3, the same set of neighbouring genes were present in these four species. We have successively a transcriptional regulator, the reannotated PTCase, an amino acid permease (probably an antiporter), a conserved hypothetical protein and finally the carbamate kinase ArcC-3 (EC 2.7.2.2). In a next step, we found that the so-called conserved hypothetical protein is homologous to the agmatine deiminase (or agmatine iminohydrolase, EC 3.5.3.12) of *Bacillus cereus *[15]. Fig. 3 further shows that the gene order found in *E. faecalis*, is completely conserved in *Lc. lactis *and *S. mutans *and slightly modified in *L. monocytogenes*.

Thus, these four clusters of genes appear to encode the full set of enzymes which are expected to form the catabolic agmatine deiminase pathway [10]. Agmatine deiminase, PTCase and carbamate kinase were already known to become coinduced by agmatine in *E. faecalis *when it is used as sole energy source [10], strongly suggesting that these gene clusters are functional operons. In *Pseudomonas aeruginosa *PAO1, the homologous agmatine deiminase is encoded by the gene *aguA *belonging to an operon *aguBA *induced by agmatine and N-carbamoylputrescine ([16,17]) but in this species N-carbamoylputrescine is converted by a N-carbamoylputrescine amidohydrolase (EC 3.5.1.53, the *aguB *product) into putrescine and CO_2 _+ ammonium rather than into putrescine and carbamoylphosphate. More recently, a similar pathway for polyamine biosynthesis has been identified by homology in higher plants [18]. In the alternative pathway corresponding to the analogous sets of genes shown on Fig. 3, it is thus a PTCase which catalyzes the second step and catabolically converts N-carbamoylputrescine to putrescine and carbamoylphosphate which can further be used to synthesize ATP via carbamate kinase [19].

Furthermore, when we compare the clusters of genes shown in Fig. 3 to those surrounding gene *argF *or *arcB*, encoding the catabolic OTCase functioning in the arginine deiminase (ADI) pathway present in many microbial genomes (see [20-22]), we observe a very similar distribution, namely a transcriptional regulator, the arginine deiminase (EC 3.5.3.6), an arginine/ornithine antiporter and a (sometimes two) carbamate kinase. There is thus a very close analogy between the set of genes encoding the enzymes catalyzing the different steps of the agmatine deiminase pathway found in these different Firmicutes and that encoding the enzymes catalyzing the different steps of the arginine deiminase pathway.

## Conclusions

Genome annotation requires both reliable tools for identifying gene function and manual expertise. The frustration due to the high percentage of orphan genes found in all genomes is often compounded with another – more vicious – problem which may occur when a very strong sequence similarity is obscuring the actual functional identity of another kind of orphan. The analysis described in this paper illustrates the difficulty in identifying such a potential source of misannotation and delineates at least two fundamental parameters which must be considered especially when the results appear to be straightforward. First, one must keep in mind that proteins sharing a high level of identical residues may have different functions. A routine step for challenging the functional annotation of any putative coding sequence should be a phylogenetic analysis. Any CDS found to branch far from its homologues in an evolutionary tree, as observed in the case of the carbamoyltransferases (Fig. 1), should be examined with caution before assigning it a putative function. Another example of the usefulness of the phylogenetic approach to correct misannotations can be found in a comparative analysis of ureohydrolases [20].

The second parameter which must be considered is the striking lack of information in the various public databases. For example, in the case studied here (the putrescine carbamoyltransferase EC 2.1.3.6) it is reported that there is no sequence available in various first-rate databases specialized in enzymatic and/or metabolic data such as ENZYME [23], BRENDA [24], KEGG [25], BIOCYC [26], etc...as well as in the Gene Ontology (GO:0050231) Consortium [27]. A significant part of this deficit of information appears to be due to not correlating biochemical data [8-11] previously published and well recorded in BRENDA [24], for example, with the incomplete amino acid sequence which was not taken into account although it had been published by the same group [13] who studied this enzyme.

The specific point we would like to stress in this paper reflects a more general gap – which is widely ignored – between the enormous quantity of information buried in the sequence data and the refined knowledge built up over several decades of studies on gene regulation and protein biochemistry (recorded in [23] to [27]). In this respect, experimentally studied proteins not correlated with sequence data also qualify as "orphans". In the present case, such a resulting gap in knowledge could be bridged only because we used the experimental approach detailed in this paper. After being alerted by the unusual topology of the phylogenetic tree (Fig. 1) and the rediscovery of the partial sequence [13], a confirmation of the reannotation as PTCases was obtained when considering their signature (Fig. 2) and the gene context (Fig. 3) which differentiate them clearly from their OTCase homologues.

The strategy we used to identify such orphan sequences could work in any other case where it is possible to collect a body of facts about experimental data, homology, unnoticed sequence data, and accurate informations about gene context. Note that we incidentally used such a strategy to annotate the genes encoding a putative agmatine deiminase in the genomes listed in Fig. 3. It is highly probable that this approach can be applied to many similar cases. Therefore, our community should feel encouraged to dig in old lab books, unpublished data buried in doctoral thesis and similar documents, in order to retrieve information crucial for correct genome annotation. Moreover, it becomes urgent to design new approaches in order to efficiently explore what has been called the "bibliome" [28]. This could help to bridge important gaps in knowledge – such as exemplified in this paper- which lead to numerous errors in genome annotation. Accordingly, it would become possible to (re)annotate conserved hypothetical proteins for which there is an apparent lack of information in the various public databases.

## Methods

### Collecting sequences

Near 450 carbamoyltransferases (ATCases and OTCases) sequences were collected from the public databases SwissProt, TREMBL and TREMBLNEW [15]. To facilitate the management of these data which are continuously growing up with the onset of new completely sequenced genomes, we assemble them in a relational database (available on request). Moreover, in the case of unpublished but completely sequenced genomes, it was often possible to recover *bona fide *sequences from specific sequencing groups sites (Joint Genome Institute [29] and Sanger Institute [30]) using either BlastP or tBlastN queries. We retained only unpublished sequences aligning along their whole length with *bona fide *carbamoyltransferases and sharing no less than 30% identity with it, using at least two distantly related seeds.

### Reconstructing phylogenetic trees

Rooted phylogenetic trees were derived from multiple alignements of ATCases and OTCases using two different approaches. (1) New sequences were manually added and aligned to the previously published [4] multiple alignement using the BioEdit sequence alignment editor [31]. These additions were made effortless by introducing each new sequence near its closest partner (the first hit in a routine BlastP check). This processive approach minimized the risk of introducing any bias when adding numerous new sequences. However, the soundness of this manual alignement was routinely checked using automatic programmes (both ClustalX and DARWIN, see below) to verify that we did not miss any conserved motifs. We further ascertained this multiple alignement (especially the introduction of gaps) by using the informations available from the known 3D structures of ATCases and OTCases. Maximum parsimony and distance trees were derived from this alignment using the PROTPARS and NEIGHBOR programmes of the PHYLIP package [32], respectively. This PHYLIP package was further used to derive confidence limits for each node of either parsimony or distance trees using a bootstrap approach (programmes SEQBOOT and CONSENSE). (2) The PhyloTree programme of the DARWIN package [33] allows to build a multiple alignement and to derive a distance tree which is an approximation to maximum likelihood tree since the deduced evolutionary distances are weighted by computing their variance when reconstructing the tree.

## Authors' contributions

NG dug up the "ancient" data on putrescine carbamoyltransferase, contributed his knowledge about carbamoyltransferases and made important additions to the manuscript. YX brought essential informations about the genetics and biochemistry of the enzymes involved in arginine metabolism and their evolution. DGN participated in the collection of new carbamoyltransferase sequences and their manual alignment and identified which sequence of *E. faecalis *is the putrescine carbamoyltransferase. BL carried out the phylogenetic analyses, the gene context study and drafted the manuscript which was further improved (and approved) by all authors.
